# Acacetin Inhibits Glutamate Release and Prevents Kainic Acid-Induced Neurotoxicity in Rats

**DOI:** 10.1371/journal.pone.0088644

**Published:** 2014-02-10

**Authors:** Tzu-Yu Lin, Wei-Jan Huang, Chia-Chan Wu, Cheng-Wei Lu, Su-Jane Wang

**Affiliations:** 1 Department of Anesthesiology, Far-Eastern Memorial Hospital, New Taipei, Taiwan; 2 Graduate Institute of Pharmacognosy, Taipei Medical University, Taipei, Taiwan; 3 Graduate Institute of Basic Medicine, Fu Jen Catholic University, New Taipei, Taiwan; 4 Department of Mechanical Engineering, Yuan Ze University, New Taipei, Taiwan; University of North Dakota, United States of America

## Abstract

An excessive release of glutamate is considered to be a molecular mechanism associated with several neurological diseases that causes neuronal damage. Therefore, searching for compounds that reduce glutamate neurotoxicity is necessary. In this study, the possibility that the natural flavone acacetin derived from the traditional Chinese medicine *Clerodendrum inerme* (L.) Gaertn is a neuroprotective agent was investigated. The effect of acacetin on endogenous glutamate release in rat hippocampal nerve terminals (synaptosomes) was also investigated. The results indicated that acacetin inhibited depolarization-evoked glutamate release and cytosolic free Ca^2+^ concentration ([Ca^2+^]C) in the hippocampal nerve terminals. However, acacetin did not alter synaptosomal membrane potential. Furthermore, the inhibitory effect of acacetin on evoked glutamate release was prevented by the Cav2.2 (N-type) and Cav2.1 (P/Q-type) channel blocker known as ω-conotoxin MVIIC. In a kainic acid (KA) rat model, an animal model used for excitotoxic neurodegeneration experiments, acacetin (10 or 50 mg/kg) was administrated intraperitoneally to the rats 30 min before the KA (15 mg/kg) intraperitoneal injection, and subsequently induced the attenuation of KA-induced neuronal cell death and microglia activation in the CA3 region of the hippocampus. The present study demonstrates that the natural compound, acacetin, inhibits glutamate release from hippocampal synaptosomes by attenuating voltage-dependent Ca^2+^ entry and effectively prevents KA-induced in vivo excitotoxicity. Collectively, these data suggest that acacetin has the therapeutic potential for treating neurological diseases associated with excitotoxicity.

## Introduction

Glutamate is the principal excitatory neurotransmitter in the central nervous system (CNS) and plays a critical role in numerous functions, such as cognition, movement, learning, and memory [1], [2]. However, in addition to the physiological role of glutamate, excessive glutamate release and activation of the glutamate receptors induce an increase in intracellular Ca^2+^ levels, which subsequently triggers a cascade of cellular responses, including enhanced oxygen free radical production, disturbed mitochondrial function, and protease activation, which ultimately kill the neurons [3], [4], [5]. This process has been implicated as a pathophysiological factor in multiple neurological disorders, both acute, such as stroke and head trauma, and chronic, such as neurodegenerative disorders [6], [7], [8]. Therefore, inhibiting the central glutamatergic neurotransmission might provide a potential strategy for treating these diseases. Consequently, several glutamatergic modulators are being developed, including N-methyl-D-aspartic acid (NMDA) receptor antagonists, and metabotropic glutamate receptor agonists and antagonists. However, these drugs have been unsuccessful in clinical trials because of numerous side effects, such as ataxia and memory impairment [9], [10]; thus, the search for new drugs that target neurological disorders continues.

**Figure 1:**
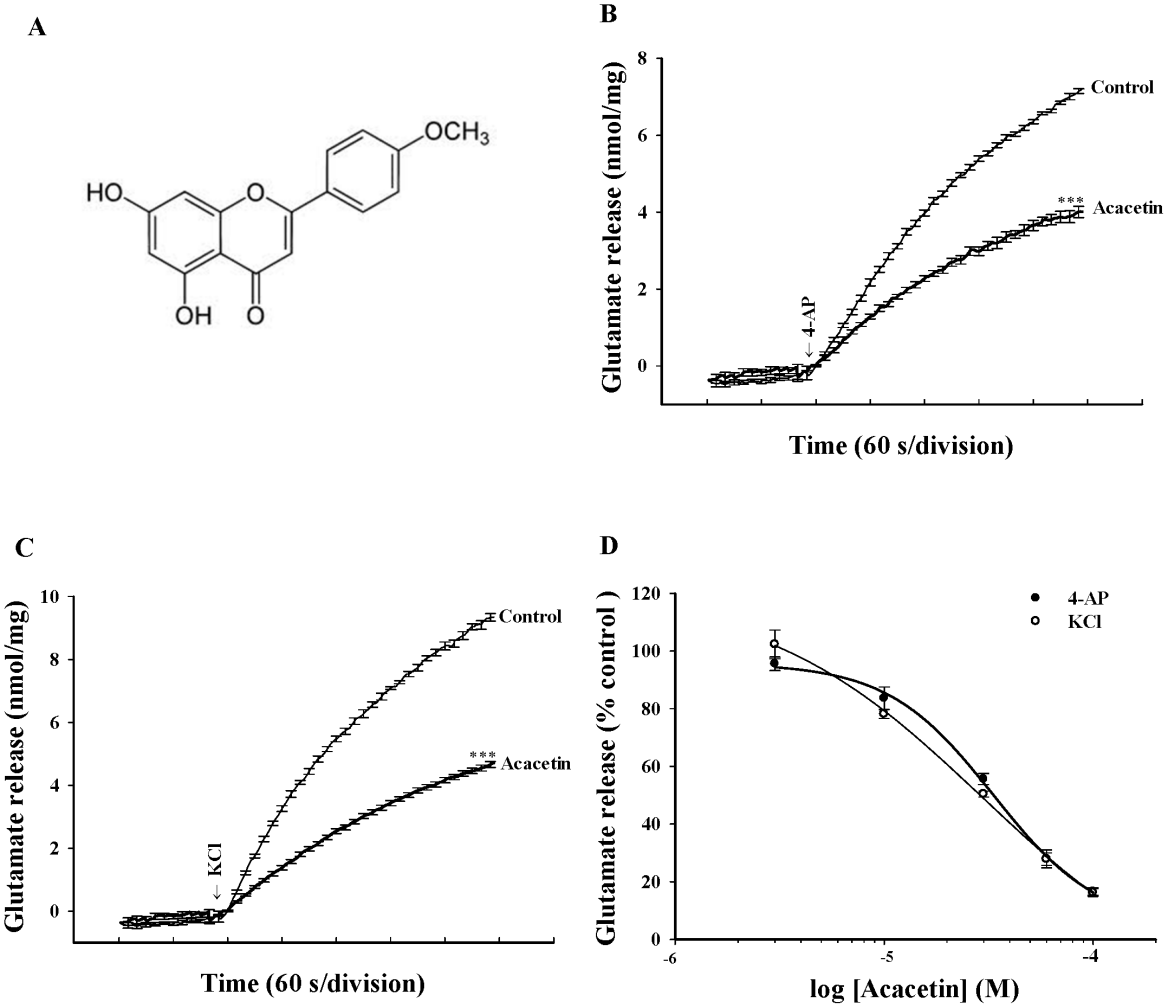
Acacetin inhibits 4-AP and KCl-evoked glutamate release from rat hippocampal nerve terminals in a concentration-dependent manner. A: Chemical structure of acacetin. B, C: Glutamate release was measured under control conditions or in the presence of 30 µM acacetin added 10 min prior to the addition of 4-AP (1 mM) or KCl (15 mM). D: Dose-response curves of decrease in 4-AP or KCl-evoked glutamate release in the presence of acacetin. Results are mean ± SEM of independent experiments, using synaptosomal preparations from six animals. ***, *P*<0.001 versus control group.

Recently, numerous studies have focused on herbal compounds that may prevent specific neurological disorders. For example, berberine (an active component of berberis), curcumin (an active component of turmeric), honokiol (an active component of magnolia officinalis), and tanshinone IIA (an active component of danshen), were able to penetrate the brain and protect it against brain damage in various animal models with neurological disorders [11], [12],[13],[14]. These plant-derived compounds also have been demonstrated to decrease the glutamate release in rat brain tissues [15], [16], [17], [18]. In the current study, we focused on acacetin (5,7-dihydroxy-4-methoxyflavone, Figure 1A), a flavonoid compound isolated from *Clerodendrum* inerme (L.) Gaertn (CI), which is a local herb that was suggested to be a potential therapeutic application for treating neuropsychiatric disorders [19]. Acacetin exhibits various biological activities including those that are anti-inflammatory, antioxidant, and anticarcinogenic [20], [21], [22]. However, the biological targets and effects of acacetin on the CNS are largely unknown, although the neuroprotective effects of acacetin on the CNS were previously suggested [23].

Considering that the excitotoxicity caused by excessive glutamate is believed to play pivotal roles under numerous neuropathological conditions [24], we investigated (1) whether acacetin affects glutamate release; and (2) whether acacetin executes a protective action against the excitotoxicity induced by kainic acid (KA), which is a glutamate analog. In the first series of experiments, we used isolated nerve terminals (synaptosomes) purified from the rat hippocampus as a model to examine the effects of acacetin on the release of endogenous glutamate, the synaptosomal plasma membrane potential, the Na^+^ influx, and the activation of the voltage-dependent Ca^2+^ channels (VDCCs). The isolated presynaptic terminals (synaptosomes) also represent a model for directly investigating the underlying molecular mechanisms of presynaptic phenomena. Specifically, synaptosomes are capable of accumulating, storing, and releasing neurotransmitters, and is devoid of functional glial and nerve cell body elements that might cause the findings to be misinterpreted because of modulatory loci at the non-neuronal, postsynaptic, or network levels [25]. In the second experimental series, we used a rat model that was treated using KA, which induced hippocampal neuronal death and microglial activation, to examine whether acacetin was effective in this model. This model was chosen because KA-induced neurodegeneration and neuronal cell death have been proposed to be linked to the pathological release of glutamate [26], [27], [28]. Therefore, administering KA to rodents is generally assumed to be an adequate model of excitotoxicity.

## Materials and Methods

### Chemicals

Acacetin was isolated from *Clerodendrum inerme*, as described previously [19]. Air dried leaves of *Clerodendrum inerme* (3.0 kg) were grinded and repeatedly extracted with 95% EtOH (10 L) for three times. The combined EtOH layers were concentrated in vacuo to give a residue (246 g). The residue was triturated with *n*-hexane, CH_2_Cl_2_, *n*-BuOH and H_2_O, respectively. CH_2_Cl_2_ layer was concentrated in vacuo to provide a residue (16 g) and then subjected to silica gel chromatography to yield fractions 1–9. One compound that was crystallized from fraction 7 using EtOH as a solvent was identified as acacetin based on NMR spectrum comparable to literature reported. The purity of the compound was more than 98% on high-performance liquid chromatography (HPLC). 3′, 3′, 3′-dipropylthiadicarbocyanine iodide [DiSC_3_(5)] and fura-2-acetoxymethyl ester (Fura-2-AM) were purchased from Invitrogen (Carlsbad, CA, USA). 4-aminopyridine (4-AP), ω-conotoxin MVIIC (ω-CgTX MVIIC), dantrolene, 7-chloro-5-(2-chloropheny)-1,5-dihydro-4,1-benzothiazepin-2(3H)-one (CGP37157), kainic acid (KA), and all other reagents were purchased from Sigma-Aldrich (St. Louis, MO). Acacetin, DiSC_3_(5), SBFI, and Fura-2 were dissolved in 0.1% dimethylsulfoxide (DMSO). 4-AP, ω-CgTX MVIIC, dantrolene, and CGP37157 were dissolved in normal saline.

### Animals

Adult male Sprague-Dawley rats (150-200 g) (n = 45) were used in this study. All of the animal experiments were performed in accordance with the National Institutes of Health Guidelines for the Care and Use of Laboratory Animals (NIH Publication No. 85-23, revised 1996), and were approved by the Fu Jen Institutional Animal Care and Utilization Committee (FJIACUC; Permit Number: A9942). All efforts were made to minimize the number of animals used and their sufferings.

### Synaptosomal preparation

Percoll-purified synaptosomes were prepared using the hippocampus of rats, as described previously [29]. The rats were sacrificed by decapitation and the brains were remove at 4°C. The hippocampus was rapidly dissected, homogenized in a medium containing 320 mM sucrose, pH 7.4. The homogenate was centrifuged at 3000 g (5000 rpm in a JA 25.5 rotor; Beckman Coulter, Inc., USA) for 10 min at 4°C, and the supernatant was centrifuged again at 14,500 g (11 000 rpm in a JA 25.5 rotor) for 12 min at 4°C. The pellet was gently resuspended in 8 ml of 320 mM sucrose, pH 7.4. Two milliliters of this synaptosomal suspension was placed into 3 ml Percoll discontinuous gradients containing 320 mM sucrose, 1 mM EDTA, 0.25 mM DL-dithiothreitol, and 3, 10 and 23% Percoll, pH 7.4. The gradients were centrifuged at 32,500 g (16 500 rpm in a JA 20.5 rotor) for 7 min at 4°C. Synaptosomes sedimenting between the 10 and the 23% Percoll bands were collected and diluted in a final volume of 30 ml of HEPES buffer medium (HBM) consisting of 140 mM NaCl, 5 mM KCl, 5 mM NaHCO_3_, 1 mM MgCl_2_⋅6H_2_O, 1.2 mM Na_2_HPO_4_, 10 mM glucose, and 10 mM HEPES (pH 7.4). Protein concentration was determined using the Bradford assay. Synaptosomes were centrifuged in the final wash to obtain synaptosomal pellets with 0.5 mg protein. The synaptosomal pellets were stored on ice and used within 4–6 h.

### Glutamate release

The glutamate release was assayed by using on-line fluorimetry, as described previously [29]. Synaptosomal pellets (0.5 mg of protein) were resuspended in HBM containing 16 µM bovine serum albumin and incubated in a stirred and thermostated cuvette at 37°C in a Perkin-Elmer LS-55 spectrofluorimeter (PerkinElmer Life and Analytical Sciences, Waltham, MA). NADP^+^ (2 mM), glutamate dehydrogenase (GDH, 50 U/mL) and CaCl_2_ (1.2 mM) were added after 3 min. After an additional 10 min of incubation, 4-AP (1 mM) or high external KCl (15 mM) was added to stimulate glutamate release. Glutamate release was monitored by measuring the increase in fluorescence (excitation and emission wavelengths of 340 and 460 nm, respectively) caused by NADPH being produced by the oxidative deamination of released glutamate by GDH. Data were accumulated at 2-s intervals. A standard of exogenous glutamate (5 nmol) was added at the end of each experiment, and the fluorescence response used to calculate released glutamate was expressed as nanomoles of glutamate per milligram of synaptosomal protein (nmol/mg). Values quoted in the text and depicted in bar graphs represent the levels of glutamate cumulatively released after 5 min of depolarization, and are expressed as nmol/mg/5 min. Estimation of the IC_50_ was based on a one-site model [Inhibition  =  (Inhibition _MAX_ × [acacetin] / (IC_50_ + [acacetin])], and calculated using the nonlinear curve-fitting function provided in MicroCal Origin. Cumulative data were analyzed using Lotus 1-2-3.

### Plasma membrane potential

The plasma membrane potential was determined using a membrane-potential-sensitive dye, DiSC_3_(5)[30]. Synaptosomes were resuspended in HBM and incubated in a stirred and thermostated cuvette at 37°C in a Perkin-Elmer LS-55 spectrofluorimeter. After 3 min of incubation, 5 µM DiSC_3_(5) were added and allowed to equilibrate before the addition of CaCl_2_ (1 mM) after 4 min of incubation. 4-AP was then added to depolarize the synaptosomes for 10 min, and DiSC_3_(5) fluorescence was monitored at excitation and emission wavelengths of 646 and 674 nm, respectively. Cumulative data were analyzed using Lotus 1-2-3 and expressed in fluorescence units.

### The cytosolic free Ca^2+^ concentration ([Ca^2+^]_C_) in the synaptosomal population

The [Ca^2+^]_C_ was measured using the Ca^2+^ indicator fura-2. Synaptosomes (0.5 mg/mL) were resuspended in HBM containing 0.1 mM CaCl_2_ and loaded with 5 µM Fura-2-AM for 30 min at 37°C. The synaptosomes were washed with HBM by being centrifuged, resuspended in 2 mL of HBM containing BSA, and placed in a Perkin-Elmer LS-55 spectrofluorometer at 37°C with stirring in the presence of 1.2 mM CaCl_2_. The synaptosomes were incubated for 10 min in the presence of acacetin (30 µM) prior to being depolarized with 4-AP (1 mM). Fura-2-Ca fluorescence was determined at excitation wavelengths of 340 and 380 nm (emission wavelength, 505 nm), and data were accumulated at 2 s intervals. [Ca^2+^]_C_ (nM) was calculated by using calibration procedures [31] and equations described previously [32]. Cumulative data were analyzed using Lotus 1-2-3.

### Cytosolic free Na^+^ concentration ([Na^+^]_C_)

Na^+^ measurements were performed in essentially the same manner as those performed in the [Ca^2+^]_C_ determinations, except the synaptosomes were preincubated with 5 µM SBFI-AM instead of fura-2 [33]). SBFI fluorescence was monitored by applying the same method used in the fura-2 experiments. The results were expressed as ratios of fluorescence (emission wavelength, 505 nm) at excitation wavelengths of 340 and 380 nm (340/380 nm).

### Histological analysis of neuronal death by neutral red and Fluoro-Jade B staining

Rats (n = 24) were randomly divided into four experimental groups: (1) control group; (2) KA-treated group; (3) KA and acacetin 10 mg/kg-treated group; and (4) KA and acacetin 50 mg/kg-treated group. Acacetin was injected intraperitoneally (i.p.) 30 min before KA (15 mg/kg; i.p.) injection. Rats were sacrificed 3 days after KA (15 mg/kg) injection by using an overdose of anesthetics (chloral hydrate, 650 mg/kg, i.p.). The animals were then perfused transcardially with saline (room temperature) followed by cold 4% paraformaldehyde in 0.1 M PBS. The brains were removed immediately and post-fixed in the same fixative overnight at 4°C, and then cryoprotected in 30% sucrose for 24–48 h. To perform neutral red staining, the brains were sectioned coronally into 30-µm-thick sections in a cryostat. The sections were then mounted on gelatin-coated slides, air dried and then stained with neutral red solution. Fluoro-Jade B (Chemicon, Millipore Ltd, Billerica, MA) staining was performed as described previously [34]. In summary, the sections (20 µm) were mounted on gelatin-coated slides and dried at room temperature followed by a solution containing 1% sodium hydroxide in 80% ethanol for 5 min. After the slides were immersed in 70% ethanol for 2 min and in distilled water for 2 min, the sections were oxidized in 0.06% potassium permanganate for 15 min, washed with water, and then immersed in 0.001% Fluoro-Jade B solution for 30 min in the dark. The slides were then washed in distilled water, air dried, cleared, and coverslipped. According to previous studies [35], [36], the hippocampus CA3 is the most vulnerable area to excitotoxic lesions caused by kainic acid. Therefore, the CA3 region was visualized under 100X magnification using an upright fluorescence microscope (Zeiss Axioskop 40, Goettingen, Germany) and digitized photomicrographs used for analysis were captured using a digital camera (Nikon D80, Tokyo, Japan) between bregma −2.30 mm and −3.60 mm according to the rat brain atlas of Paxino and Watson [37]. To compare neuronal death among the experimental groups, the number of Fluoro-Jade B-positive cells was measured in a 255×255 µm area of the hippocampal CA3 in 6 to 8 randomly chosen sections from each animal and averaged for each animals using a computer-assisted image analysis system (Image J; NIH Image, National Institutes of Health, Bethesda, MD, USA) by an examiner blind to experimental conditions. Results were expressed as mean ± SEM of labeled cells per 0.1 mm^2^.

### Immunohistochemistry

Fixed brains were cut into 40-µm-thick coronal sections in a cryostat and then free-floating staining was performed using an immunohistochemical ABC method. In summary, after rinsing the sections 3 times with PBS, the sections were blocked with 2% normal goat serum containing 0.3% Triton X-100 for 1 h at room temperature. The sections were then incubated overnight at 4°C with a mouse monoclonal anti-OX-42 antibody (1∶500; Santa Cruz Biotechnology Inc). The sections were then incubated with a goat biotinylated anti-mouse secondary antibody (1∶200; Vector Laboratories, Burlingame, CA) for 2 h, and subsequently incubated with ExtrAvidin peroxidase (1∶1000, Sigma-Aldrich) for 1 h at room temperature. After rinsing the sections in 0.1 M PBS for 20 min, the sections were reacted with 0.025% 3,3′-diaminobenzidine tetrahydrochloride (DAB) solution in PBS containing 0.0025% hydrogen peroxide for 6 min. The sections were then mounted on gelatin-coated glass slides, air-dried, dehydrated, cleared with xylene, and coverslipped with Entellan mounting medium (Merck, Darmstadt, Germany).

### Statistical analysis

Data were expressed as mean ± SEM. The data reported were analyzed by using the unpaired Student's *t* test or by using one-way ANOVA accompanied by post-hoc LSD comparison tests for multiple comparisons. The analysis was completed using SPSS software (17.0; SPSS Inc., Chicago, IL). *P*<0.05 was considered to represent a significant difference.

## Results

### Acacetin inhibits evoked glutamate release in rat hippocampal synaptosomes

In the first set of experiments, isolated nerve terminals were depolarized using the potassium channel blocker 4-AP or high external [K^+^] to investigate the effect of acacetin on glutamate release. In synaptosomes incubated in the presence of 1 mM CaCl_2_, 4-AP (1 mM) evoked a glutamate release of 7.2±0.1 nmol/mg/5 min. The application of acacetin (30 µM) decreased the amount of 4-AP-evoked glutamate release to 4.0±0.1 nmol/mg/5 min (*P*<0.001) without altering the basal release of glutamate (Figure 1B). Similarly, the release of glutamate evoked by KCl (15 mM) was also inhibited in the presence of acacetin (30 µM; *P*<0.001; Figure 1C). The acacetin-mediated inhibition of 4-AP- or KCl-evoked glutamate release was concentration dependent, and produced an IC50 value of approximately 31 µM and 29 µM, respectively, which was derived from a dose-response curve (Figure 1D).

### Acacetin reduces the depolarization-induced increase in [Ca^2+^]_C_ but does not alter the synaptosomal membrane potential

**Figure 2:**
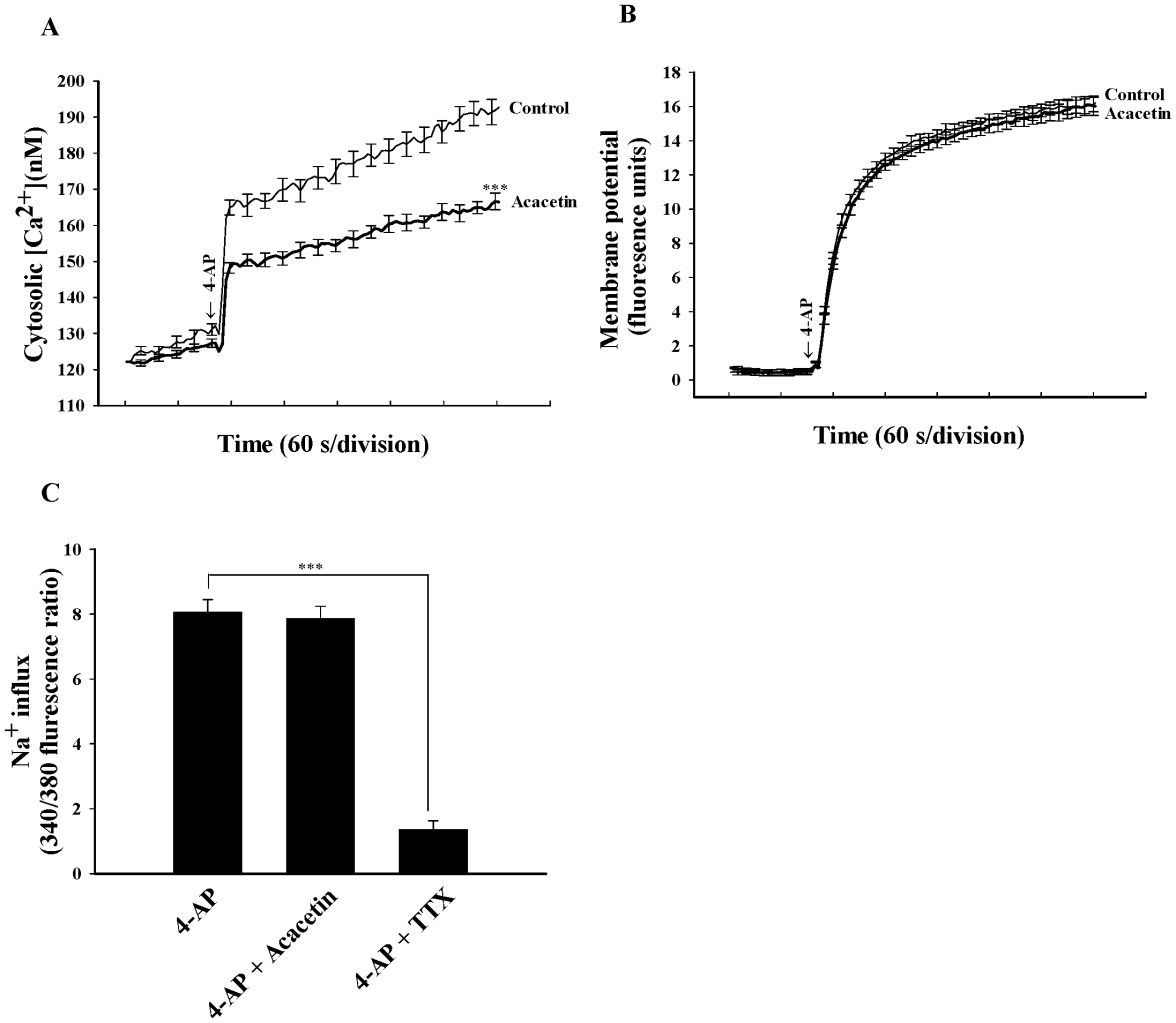
Acacetin reduces intrasynaptosomal Ca^2+^ levels but does not alter the synaptosomal membrane potential and Na^+^ influx. Cytosolic free Ca^2+^ concentration (nM) (A) or synaptosomal membrane potential (B) was measured in the absence (control) and in the presence of 30 µM acacetin, added 10 min before depolarization with 1 mM 4-AP. C: Na^+^ influx was induced by 1 mM 4-AP in the absence (control) or presence of 30 µM acacetin or 2 µM TTX, added 10 min before depolarization. Results are mean ± SEM of independent experiments, using synaptosomal preparations from five animals. ***, *P*<0.001 versus control group.

Transmitter release can be modulated by regulating the plasma membrane potential, and consequently altering the calcium influx. To investigate the potential mechanisms underlying the acacetin-mediated inhibition of glutamate release, the effect of acacetin on intrasynaptosomal Ca^2+^ levels was determined by using the Ca^2+^ indicator Fura-2. In Figure 2A, 4-AP (1 mM) caused a rise in cytosolic Ca^2+^ concentration ([Ca^2+^]_C_) from 127.1±1.2 nM to a plateau level of 196.8±5.5 nM (*P*<0.001). Applying acacetin (30 µM) did not affect basal Ca^2+^ levels, but caused an approximately 18% decrease in the 4-AP-evoked rise in [Ca^2+^]c (161.5±4.6 nM; *P*<0.001; Figure 2A). The inhibition of the [Ca^2+^]_C_ elevation by acacetin might be attributed either to a direct reduction in the amount of Ca^2+^ entering through VDCCs, or to secondary effects caused by, for example, the modulation of potassium channels and the consequently altered plasma membrane potential. To discern between these two possibilities, the effect of acacetin on the synaptosomal plasma membrane potential under resting conditions and on depolarization was examined using membrane potential-sensitive dye DiSC_3_(5). Figure 2B demonstrates that 4-AP (1 mM) caused an increase in DiSC_3_(5) fluorescence of 16.3±0.8 fluorescence units/5 min. Preincubation of the synaptosomes using acacetin (30 µM) for 10 min before adding 4-AP did not alter the resting membrane potential, and produced no substantial change in the 4-AP-mediated increase in DiSC_3_(5) fluorescence (16.1±0.6 units/5 min; *P* = 0.812). In addition, the Na^+^-sensitive probe SBFI was used to measure cytosolic Na^+^ levels. Figure 2C indicates that 4-AP (1 mM) caused a clear rise in Na^+^ influx, but acacetin (30 µM) failed to affect this increase (*P* = 0.792). The failure of acacetin to produce an effect on this increase was not caused by an insufficient level of sensitivity of the SBFI probe to alterations in Na^+^ channel activity because in parallel experiments, the Na^+^ channel blocker tetrodotoxin (TTX; 2 µM) caused an 82% inhibition of 4-AP-evoked Na^+^ influx (Figure 2C; *P*<0.001). These results indicate that the observed effect of acacetin on [Ca^2+^]_C_ is most likely caused by a direct modulation of VDCCs activity.

### Involvement of presynaptic N- and P/Q-type Ca^2+^ channels in the acacetin-mediated inhibition of glutamate release

**Figure 3:**
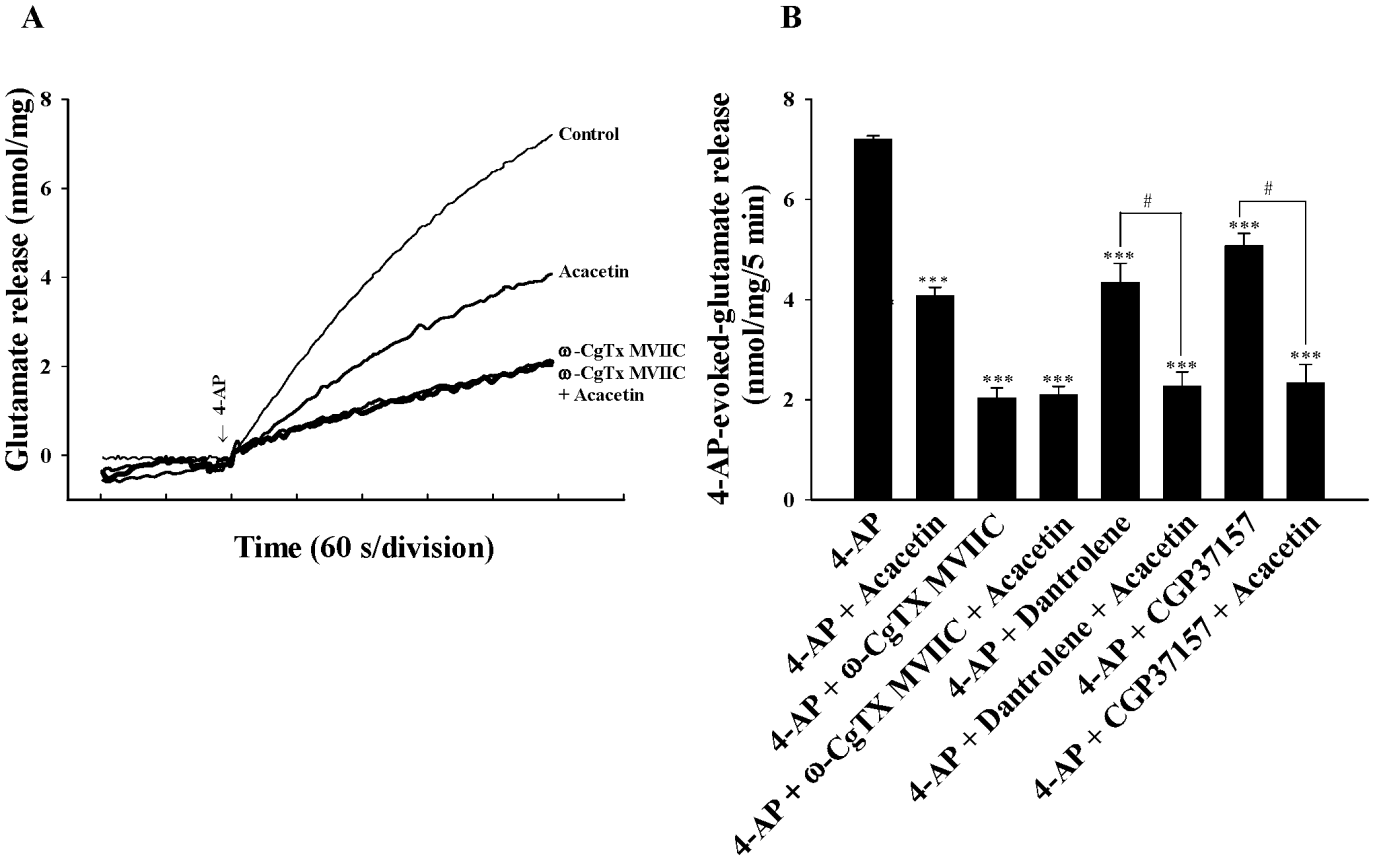
Acacetin-mediated inhibition of glutamate release is prevented by blocking the Ca_v_2.2 (N-type) and Ca_v_2.1 (P/Q-type) channels. A: Glutamate release was evoked by 1 mM 4-AP in the absence (control) or presence of 30 µM acacetin, 2 µM ω-CgTX MVIIC, 2 µM ω-CgTX MVIIC and 30 µM acacetin. B: Quantitative comparison of the extent of glutamate release by 1 mM 4-AP in the absence or presence of 30 µM acacetin, and absence and presence of 2 µM ω-CgTX MVIIC, 100 µM dantrolene, or 100 µM CGP37157. Results are mean ± SEM of independent experiments, using synaptosomal preparations from five to six animals. ***, *P*<0.001 versus control group; #, *P*<0.05 versus dantrolene-, or CGP37157-treated group.

In the adult rat cerebrocortical nerve terminals, the release of glutamate evoked by depolarization is supported by Ca_v_2.2 (N-type) and Ca_v_2.1 (P/Q-type) channels [38], [39], [40]. To determine whether the decrease in Ca^2+^ channel activity was involved in the effect of acacetin on 4-AP-evoked glutamate release, we examined the effect of acacetin in the presence of ω-conotoxin MVIIC (ω-CgTX MVIIC), a wide spectrum blocker of Ca_v_2.2 (N-type) and Ca_v_2.1 (P/Q-type) Ca^2+^ channels. In Figure 3A, 4-AP (1 mM)-evoked glutamate release (7.2±0.3 nmol/mg/5 min) was substantially reduced in the presence of ω-CgTX MVIIC (2 µM) (2.0±0.2 nmol/mg/5 min; *P*<0.001). Although the 4-AP-evoked glutamate release was considerably reduced in the presence of acacetin (30 µM), this effect was prevented by the presence of ω-CgTX MVIIC. The release measured in the presence of both ω-CgTX MVIIC and acacetin was similar to that obtained in the presence of ω-CgTX MVIIC alone [F(2,13) = 413.856, *P* = 0.792] (Figure 3A and 3B). In addition to the Ca^2+^ influx through VDCCs, the release of glutamate evoked by depolarization was reported to be caused by a Ca^2+^ release from intracellular stores such as endoplasmic reticulum (ER) and mitochondria [41]. Therefore, a potential role of intracellular Ca^2+^ release in the acacetin-mediated inhibition of glutamate release was tested in the presence of dantrolene, an inhibitor of intracellular Ca^2+^ release from endoplasmic reticulum, and CGP37157, a membrane-permeant blocker of mitochondrial Na^+^/Ca^2+^ exchange. Figure 3B indicates that 4-AP (1 mM)-evoked glutamate release was reduced by dantrolene (100 µM) (4.3±0.4 nmol/mg/5 min; *P*<0.001), indicating that the Ca^2+^ release from ER ryanodine receptors contributes substantially to the levels of 4-AP-evoked glutamate release. In the presence of dantrolene, however, acacetin (30 µM) still effectively inhibited 4-AP-evoked glutamate release [F(2,13) = 95.346, *P*<0.05]. Compared to the effects of dantrolene, CGP37157 (100 µM) similarly decreased the levels of 4-AP-evoked glutamate release, but it produced no effect on the acacetin-mediated inhibition of 4-AP-evoked glutamate release [F(2,13) = 104.843, *P*<0.05] (Figure 3B).

### Acacetin pretreatment reduces KA-induced neuronal cell death and microglial activation in the CA3 region of the hippocampus

**Figure 4:**
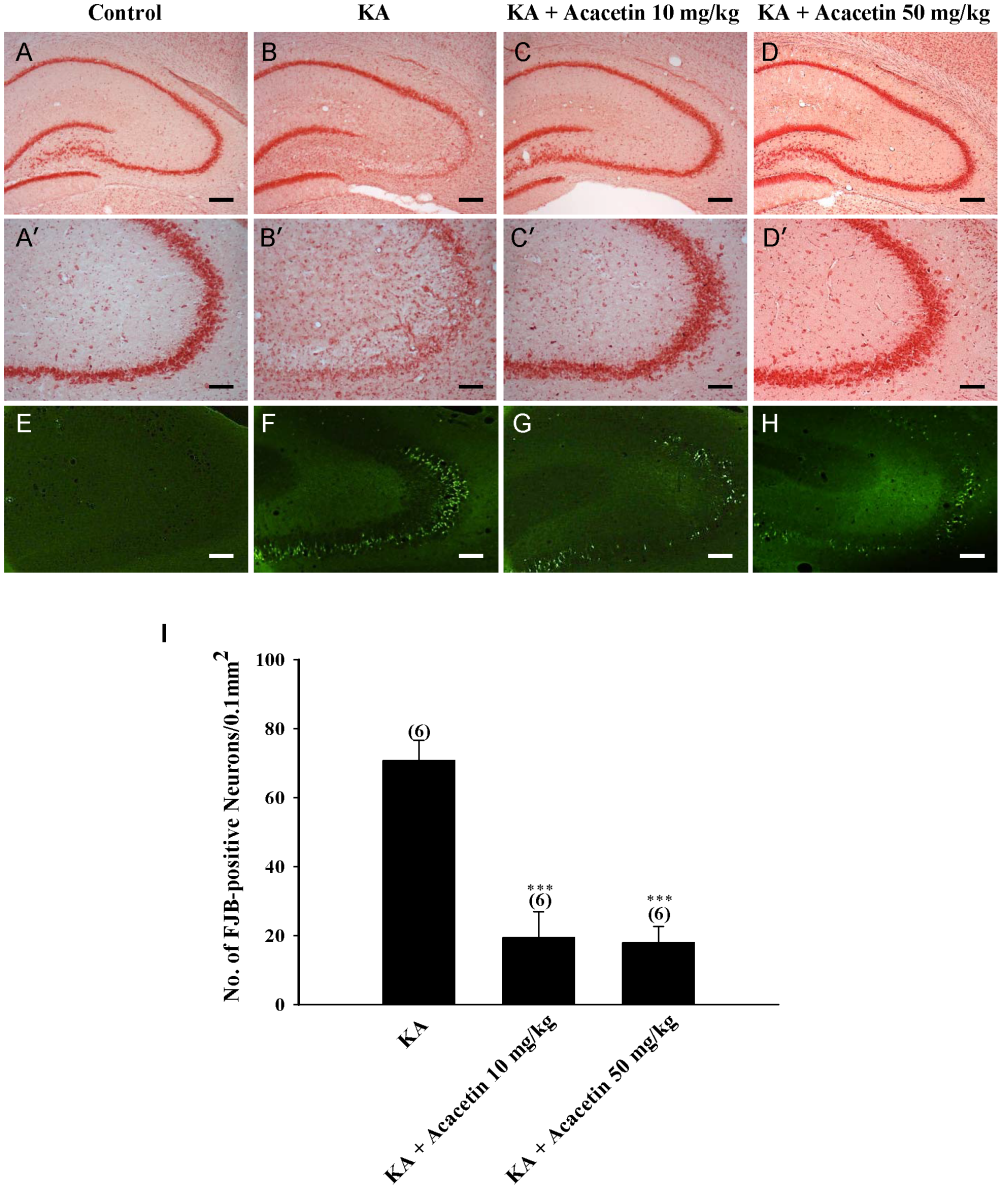
Acacetin attenuates KA-induced neuronal cell death in the CA3 region of the hippocampus. Acacetin (10 or 50 mg/kg) was administrated intraperitoneally 30 min before KA injection, and extents of neuronal losses in the hippocampus were evaluated at 3 days after KA injection by staining with neutral red (A-D) and Fluoro-Jade B (E-H). Representative photomicrographs illustrating neuronal cell death in the hippocampal CA3 region of control, KA, KA + acacetin 10 mg/kg, and KA + acacetin 50 mg/kg. (I) Quantification of Fluoro-Jade B-positive neurons in the CA3 region of the hippocampus. Data are expressed as mean ± SEM of six independent experiments. ***P<0.001, as compared with the KA-treated group. Scale bar for A-D, E-H = 250 µm, A′-D′ = 100 µm.

An excessive release of glutamate was implicated in the pathogenesis of acute and chronic brain disorders [24]. The experiments performed in this study demonstrated that acacetin inhibits glutamate release from hippocampal nerve terminals, thus supporting the hypothesis that acacetin produces a neuroprotective effect against exocytotoxic insults. To confirm this hypothesis, we examined the effect of acacetin on neuronal death induced by kainic acid (KA), an excitotoxic substance. The neuronal death that occurred after KA administration (15 mg/kg, i.p., 72 h) was verified using neutral red and Fluoro-Jade B staining. As displayed in Figure 4A and B, neutral red staining indicated a significant neuronal loss in the hippocampal CA3 and CA4 of KA-injected rats compared with that of the DMSO-treated rats (control). Acacetin administration (10 or 50 mg/kg, i.p.) performed 30 min before KA administration substantially reduced KA-induced neuronal death in CA3 and CA4 (Figure 4C and D). A similar protective effect of acacetin against neuronal death was observed by using Fluoro-Jade B staining. As illustrated in Figure 4E, no staining was observed in the DMSO-injected rats (control). KA treatment caused a substantial increase in the number of Fluoro-Jade B-positive neurons in the CA3 region of the hippocampus (*P*<0.001; Figure 4G and I). In rats pretreated with acacetin (10 or 50 mg/kg), the number of KA-induced degenerative neurons in CA3 was substantially reduced [F(2,15) = 24.158, *P*<0.05](Figure 4G-I).

**Figure 5:**
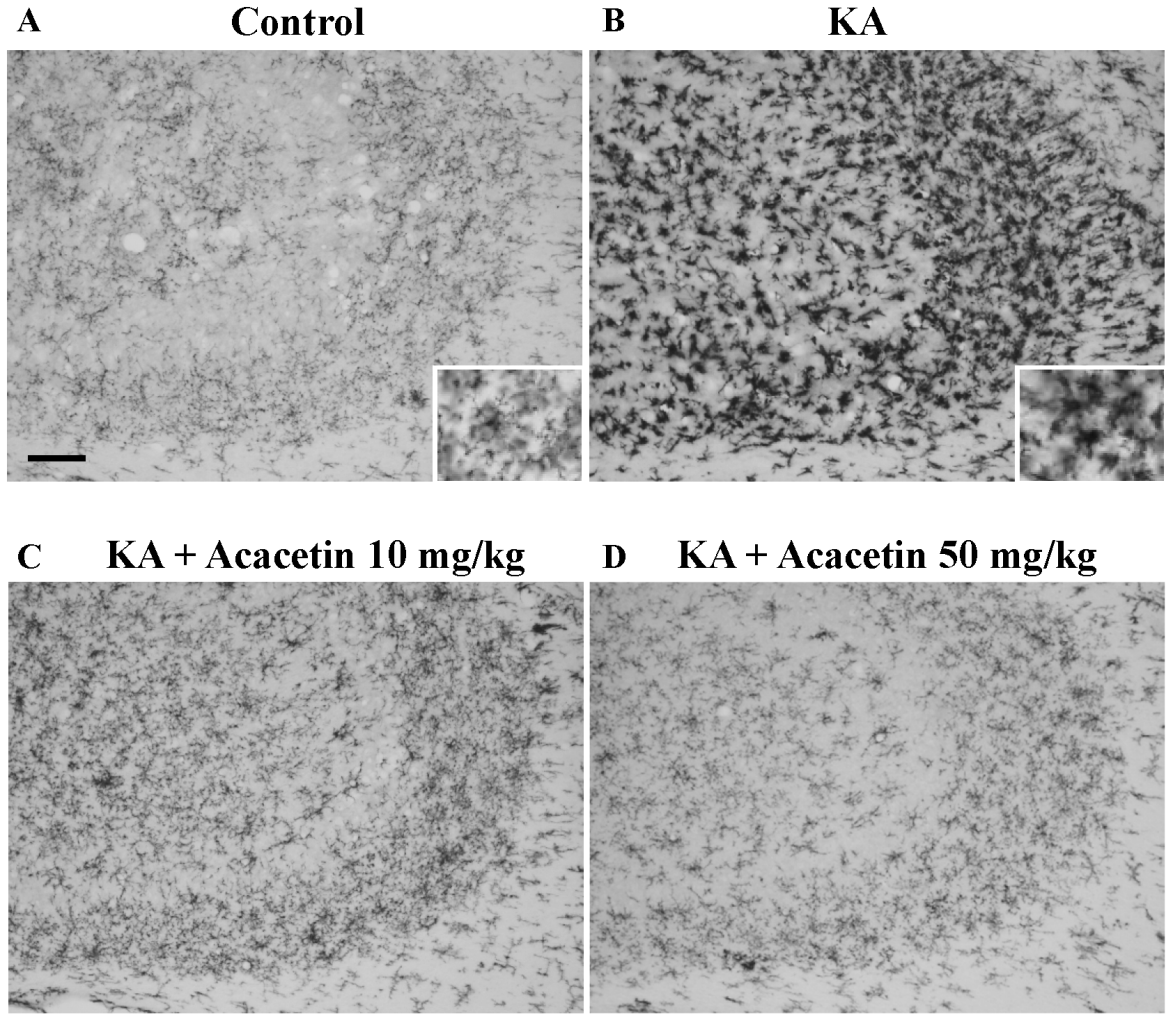
Acacetin suppresses KA-induced microglial activation in the CA3 region of the hippocampus. Acacetin (10 or 50 mg/kg, i.p.) was administrated 30 min before KA injection, and the hippocampal sections were stained with anti-OX-42 antibody at 3 days after KA injection. Representative photomicrographs illustrating OX-42 immunoreactivity in the hippocampal CA3 region of control, KA, KA + acacetin 10 mg/kg, and KA + acacetin 50 mg/kg. Insets in figure show the morphological changes after KA administration under higher magnification. Representative picture from six independent experiments were presented. Scale bar  = 100 µm for A-D.

KA-induced hippocampal neuronal death is accompanied by the increased activation of the microglia [42], [43]. To examine whether acacetin affected inflammatory processes in KA-injected brains, the activation of microglia after administering KA (72 h) was analyzed by detecting the expression of OX42, a surface marker used for microglia. In the DMSO-treated rats (control), microglial cells in the CA3 region exhibited a resting morphology with small cell bodies and thin processes (Figure 5A). Conversely the number of microglial cells in KA-injected rats increased remarkably in the CA3 region. These cells displayed enlarged cell bodies with considerably shorter and thicker processes (indicating the activation state; Figure 5B). KA-induced microglial activation was substantially suppressed in the rats pretreated with acacetin (10 or 50 mg/kg); most of the microglial cells were in a ramified or resting state (n = 6; Figure 5C and 5D).

## Discussion

Excessive glutamate release is an underlying cause of neuronal damage in a variety of CNS diseases including cerebral ischemia, epilepsy and neurodegenerative disorders [7], [8], [24]. As a result, reducing glutamate release may have critical consequences and may be a potential mechanism of neuroprotective drugs. Natural products derived from medicinal herbs have recently received a considerable amount of attention because of the beneficial effects of these products on the CNS, specifically, the neuroprotective effects. Experiments performed on animals revealed that acacetin, a naturally occurring flavonoid, plays a neuroprotective role [23]. However, the detailed mechanism of this role remains unresolved. Therefore, the purpose of this study was to enhance the understanding of the mechanism responsible for the neuroprotective effect of acacetin produced in response to excitotoxic insults. By considering both in vitro glutamate release and in vivo KA-induced excitotoxicity, we discovered that acacetin preferentially inhibits glutamate release evoked from rat hippocampal nerve terminals, and attenuates KA-induced neuronal cell death and microglia activation in the CA3 region of the hippocampus. This is the first study to assess the possible influence of acacetin on glutamate release at the presynaptic level, and on glutamate-induced excitotoxicity.

The release of glutamate from a presynaptic site is a possible target for the drug modulation of excitability and synaptic transmission in central neurons [44]. Therefore, one purpose of this study was to investigate the relationship between acacetin and the presynaptic modulation of glutamate release, and to determine the underlying molecular mechanisms. By preparing nerve terminals from rat hippocampi, we discovered that acacetin inhibited depolarization-evoked glutamate release. However, acacetin did not affect the basal release of glutamate from the nerve terminals, suggesting that acacetin might reduce the release of glutamate when it is triggered by neuronal activation.

In nerve terminals, the inhibition of Na^+^ channels or activation of K^+^ channels stabilizes membrane excitability and, consequently, causes a reduction in the levels of Ca^2+^ entry and neurotransmitter release [45], [46], [47]. The observed inhibitory effect of acacetin on evoked glutamate release could occur through a reduction of nerve terminal excitability, but this is unlikely because of the following 2 reasons: First, acacetin inhibited the release of glutamate evoked by 4-AP and KCl. Although 4-AP-evoked glutamate release involves the activation of Na^+^ and Ca^2+^ channels, 15 mM external KCl-evoked glutamate release involves only Ca^2+^ channels [48], [49], and this indicates that Na^+^ channels are not involved in the effect of acacetin on glutamate release. This suggestion was supported by the observation that acacetin did not affect the 4-AP-evoked Na^+^ influx; Second, no substantial acacetin effect on synaptosomal plasma membrane potential was observed, which indicated a lack of effect on the K^+^ conductance. These results suggest that the decreased glutamate release caused by acacetin is not the result of a reduction in synaptosomal excitability caused by ion channel (e.g., the Na^+^ or K^+^ channels) modulation. This finding disagrees with previous electrophysiological studies, which have shown that acacetin inhibits K^+^ currents in human atrial myocytes and HEK 293 cells [50], [51]. The reason for this discrepancy between the current and previous in vitro studies is unclear, but may be related to the different experimental models applied.

Therefore, if the effect is not caused by the modulation of synaptosomal excitability, then acacetin possibly inhibits evoked glutamate release by decreasing the levels of Ca^2+^ entry through the Ca_v_2.2 (N-type) and Ca_v_2.1 (P/Q-type) Ca^2+^ channels that are coupled to glutamate exocytosis in the nerve terminals [38], [39]. This hypothesis is plausible because we demonstrated that acacetin decreased the 4-AP-evoked increase in [Ca^2+^]_C_. In addition, the inhibitory effect of acacetin on glutamate release was prevented by ω-CgTX MVIIC, a wide spectrum blocker of the Ca_v_2.2 (N-type) and Ca_v_2.1 (P/Q-type) Ca^2+^ channels. However, neither dantrolene, an inhibitor of intracellular Ca^2+^ release from the endoplasmic reticulum ryanodine receptors, nor CGP37157, a mitochondrial Na^+^/Ca^2+^ exchange blocker, affected the inhibitory effect of acacetin on glutamate release. Based on these results, we suggest that acacetin inhibits evoked glutamate release by reducing presynaptic Ca^2+^ influx rather than by indirectly affecting neuronal excitability.

We hypothesized that acacetin would have a neuro-protective effect because we discovered that acacetin depressed glutamate release from nerve terminals, and that the excessive release of glutamate is a critical element in the neuropathology of acute and chronic brain disorders. This hypothesis was confirmed in this study by applying the KA model to adult rats. KA is an analogue of glutamate, and the systemic administration of KA to animals causes neuronal cell death or neurodegeneration in specific brain regions, such as the hippocampus, piriform cortex, thalamus, and amygdala [52]. Furthermore, previous studies have suggested that KA-induced neuronal death is linked to the pathological release of glutamate [26], [27], [28]. Therefore, administering KA to rodents is generally assumed to be an adequate method of excitotoxicity. In this study, administering KA (15 mg/kg, i.p.) caused a considerable neuronal death in the CA3/CA4 hippocampus area. This result is consistent with the results obtained when other groups applied the same dose of KA [35], [53]. In the hippocampus, CA3 pyramidal cells are most severely damaged after KA administration [35], [36], [54]. Such a phenomenon may be attributed to the highest density of KA receptors in the CA3 region [55]. In this study, KA-induced hippocampal neuronal death was also attenuated by acacetin pretreatment (10 or 50 mg/kg), suggesting that acacetin acts as a neuroprotective agent. This suggestion is in line with the results of a previous study that demonstrated that acacetin protects dopaminergic neurons against neurotoxicity in experimental models [23]. In addition, KA is an agonist for a subtype ionotropic glutamate receptors, KA and AMPA (α-amino-3-hydroxy-5-methylisoxazole-4-propioni acid) receptors. Overstimulation of these receptors is believed to contribute to KA-induced neurodegeneration [26], [27]. Thus, the present study does not exclude the possibility that the inhibition of KA-induced neuronal cell death by acacetin is linked to antagonism at the KA/AMPA receptor.

Numerous studies have indicated that KA-induced neuronal death is associated with microglia activation in the hippocampus [42], [43]. The prevention of microglia activation protects the hippocampus from neuro-degeneration caused by KA [56]. In our study, we also observed that the amount of activated microglia immunostained by OX-42 antibodies in the hippocampus was substantially higher in KA-treated rats. Acacetin pretreatment reduced the amount of activated microglia. Therefore, acacetin can be supposed to have an anti-inflammatory function, and that this action underlies, at least in part, its protective action against the excitotoxicity induced by KA. This is consistent with previous studies, which showed that acacetin protects neurons against neurotoxin- and ischemia-induced toxicity by inhibiting microglial activation and pro-inflammatory cytokine production [23],[57]. However, the manner in which acacetin affected KA-induced microglial activation is not clear. Neuroinflammation is included in the pathogenesis of numerous acute and chronic neurological disorders [58], [59]. Inflammatory processes, including microglial activation and the consequent production of various neurotoxic factors (including free radicals and pro-inflammatory cytokines), are believed to assist in causing KA-induced neuronal death [60]. Therefore, future research should determine whether acacetin affects the production of these factors in KA animal model.

Studies have reported that acacetin, at 10-25 mg/kg, protects neurons against insults induced by 1-methyl-4-phenyl-1,2,3,6-tetrahydropyridine (MPTP) and ischemia [23], [57]. However, the neural protective effects of acacetin are also reported at a lower concentration range (i.e., 50–200 nM) [23]. In the present study, 30 µM of acacetin inhibits evoked glutamate release in vitro, and acacetin at 10–50 mg/kg (approximately 50–250 µM) attenuates in vivo KA-induced neuronal death and microglia activation in the hippocampus. Although the dose of acacetin used in our present study to produce the effect was higher, the action of acacetin was specific. The observation supporting this statement revealed the following: (1) acacetin decreased the depolarization-induced increase in [Ca^2+^]_C_, whereas it did not alter 4-AP-mediated depolarization and Na^+^ influx; (2) acacetin-mediated inhibition of glutamate release was abolished by the N-, P- and Q-type Ca^2+^ channel blocker, but not by the ryanodine receptor blocker, or the mitochondrial Na^+^/Ca^2+^ exchanger blocker.

In conclusion, this study is the first to demonstrate that acacetin inhibits glutamate release from hippocampal nerve terminals in rats. This may contribute to the substantial neuroprotective effect of acacetin against KA-induced in vivo excitotoxicity. Although, the relevance of our finding to *in vivo* clinical situations remains to be determined, this investigation enhances the understanding of acacetin action in the brain and demonstrates the therapeutic potential of this natural compound in treating neurological disorders in which excitotoxic neuronal cell death and inflammation processes are involved.
